# Effects of Exposure to Formal Aquatic Activities on Babies Younger Than 36 Months: A Systematic Review

**DOI:** 10.3390/ijerph20085610

**Published:** 2023-04-21

**Authors:** Carlos Santos, Carolina Burnay, Chris Button, Rita Cordovil

**Affiliations:** 1 Faculdade de Motricidade Humana, Universidade de Lisboa, Lisboa, 1499-002 Cruz Quebrada, Portugal; 2 Interdisciplinary Center for the Study of Human Performance (CIPER), Faculdade de Motricidade Humana, Universidade de Lisboa, Lisboa, 1499-002 Cruz Quebrada, Portugal; 3 School of Physical Education, Sport and Exercise Sciences, University of Otago, Dunedin 9016, New Zealand

**Keywords:** baby swimming, infant aquatic therapy, child development, child health, child safety

## Abstract

This systematic review investigated the possible effects of exposing infants to formal activities in aquatic environments. A literature search of eight databases was concluded on 12 December 2022. Studies were eligible if they: (i) focused on 0–36 months of age infants, (ii) addressed the exposure of infants to formal aquatic activities, and (iii) compared the ‘same condition of aquatic exposure with the control’ or ‘before and after exposure’. The PRISMA protocol was used. Articles considered for inclusion (*n* = 18) were clustered in the health, development, and physiological outcome domains. The results show that research is focused on indoor activities, mainly in baby swimming programs and baby aquatic therapy interventions. Swimming and aquatic therapy practices are generally safe for babies’ health, and there are benefits to preterm and newborns exposed to aquatic therapy once the physiological parameters are maintained in normal and safe patterns. A positive effect is also suggested in general gross and fine motor skills, visual motion perception, cognitive flexibility, and response selection accuracy for infants who participated in aquatic programs. Further investigation with high-quality experimental designs is required to establish the effect of exposure of infants to formal aquatic activities (Systematic Review Registration: CRD42021248054).

## 1. Introduction

Young children aquatic activities seem to be an ancestral practice, with reports dating back to the 19th century when Western explorers first met natives of the Polynesian Islands [1]. In many Western societies, the formal exposure of babies to swimming pools is linked to the 1960s as a result of the post-World War II period, along with rapid economic development, an increase in the attention paid to early childhood education, and the improvement of facilities. Since then, babies’ formal activities in aquatic environments (e.g., baby swimming programs, aquatic therapy, or any other type of formal activity that takes place in a body of water) have spread globally.

Baby swimming programs are an extremely popular way of promoting the adaptation of young children to water environments in Western countries [2,3]. These programs can be a one-to-one intervention with the focus on self-rescue or survival skills, an alignment of exercises performed in a group routine, or a more ‘free-to-explore’ dynamic in a parent–child playful environment with toys and aquatic playgrounds, as well as other formats with a therapeutic focus on children with neuromotor impairments [4]. Another form of organized aquatic activities experiencing rapid growth are Baby SPA programs (SPA; from Latin, *salus per aquam*, meaning health through water). These programs combine massage and movements in the water in the first year of babies’ lives, with the aim of eliminating fatigue and boredom, delivering a feeling of calm and comfort so that the baby will relax and sleep soundly, and gaining inherent benefits for growth and development [5].

Alongside the rapid popularization of these types of formal aquatic activities, some scientific and pedagogical concerns have been raised [4]. Indeed, contradictory outcomes on the effects of exposing babies to formal aquatic activities have appeared. For instance, one of the first studies addressing the effects of exposing babies to swimming lessons was the classic Johnny and Jimmy study conducted by McGraw [6]. In this study, the author challenged the maturational theory by showing that the exposure of one identical twin (Johnny) to motor stimulation promoted a more rapid development of motor competencies (compared to Jimmy). Specifically, the exposure to intensive swimming practices helped Johnny to adopt swimming-associated movements earlier than Jimmy. However, in a later cross-sectional observational study, McGraw [7] reported the existence of different phases of both of the infants’ aquatic behavior, implying a stronger maturational effect (over the experiential effect).

Another long ongoing debate with ambiguous outcomes is the effect that exposing infants to formal swimming lessons may have on drowning statistics. Drowning has been identified as a public health priority by the World Health Organization [8], and children 1–4 years of age have been recognized as the most represented age range in drowning rates worldwide [9]. Considering the lack of available data to determine the effect of swimming programs on babies’ likelihood of drowning, the American Academy of Paediatrics (AAP) [10,11] initially suggested that swimming programs would not decrease the risk of drowning among young children. On the contrary, swimming lessons could offer parents a false sense of security that could lead to a less careful supervision. Later, in light of a published case–control study showing that children 1–4 years old who participated in swimming lessons were 88% less likely to engage in drowning incidents than the matching control group [12], the AAP [13] reviewed the recommended age to initiate swimming lessons, suggesting that, at any age, the participation in swimming courses could be beneficial to young children, as long as the multilayered protection recommendations were assured (i.e., adult supervision and pool barriers). Although Brenner et al.’s [12] case–control study had a wide odds ratio for children under 5 years of age, they were able to acknowledge that swimming lessons do not increase the risk of drowning for this age group.

There is also an additional concern about the effects of aquatic exposure on babies’ health (e.g., gastrointestinal tract infections, dermatitis, and acute respiratory illness) as a result of their exposure to the chemical treatment of the water [14]. A discrepancy of results was observed when analyzing studies focusing on the exposure of infants to these byproducts [15,16], maybe due to different target ages and, in some cases, to the limitations in the study designs. Nevertheless, to our knowledge, no previous review of the literature has focused on the effects that exposing babies younger than 36 months of age to aquatic programs can have on their health.

Previous systematic reviews have shown that the exposure of babies to aquatic environments positively influences babies’ neurodevelopment [17] and, also, that swimming programs are a potential way of preventing children 2–4 years old from drowning [18,19]. However, such studies have been limited by a lack of consistency in measured outcomes and in the rigor of the methods employed [18], leading to the suggestion that further investigation is required [19].

In the infant aquatic therapy field, the need for further scientific evidence is even more evident. A 2006 systematic review on the effects of aquatic interventions in 0–18 year old children with neuromotor impairments [20] found only two articles, both with a low level of evidence (i.e., one case report and a sample size of 3, without a control group).

Collectively, the existing literature has addressed the effectiveness of aquatic competence on children’ drowning prevention [18,19] and the effect of aquatic interventions on children motor impairment [20,21] and motor development [17] via systematic reviews. Yet, individually, these systematic reviews have focused on large age groups and did not consider the specific ages of 0–36 months. The current study aimed to identify and critically analyze the existing literature on the effects of exposing 0–36 months old babies to formal aquatic activities on their affective, social, cognitive, and motor development, as well as on the babies’ health and safety.

## 2. Materials and Methods

This systematic review follows PRISMA^®^ (Preferred Reporting Items for Systematic Reviews and Meta-Analyses) statement guidelines [22]. The review protocol was registered in PROSPERO with ID number CRD42021248054. PICO’s model for the definition of inclusion criteria was used. To be eligible for inclusion, articles were required to (i) focus on babies 0–36 months of age; (ii) address the exposure of babies to formal aquatic activities (i.e., activities supervised or taught by a qualified practitioner, as opposed to informal experiences provided by friends or family members, or focusing purely on hygiene purposes); and (iii) compare the ‘same condition of aquatic exposure with the control’ or ‘before and after exposure’. The outcome was deliberately broad and not specified, as the aim of this systematic review was to identify any possible outcomes arising from exposing infants to formal activities in aquatic environments. Study designs considered were full text, original, peer-reviewed studies. Experimental or quasi-experimental studies with no control group were not considered.

Literature published in English, Portuguese, and Spanish up to 12 December 2022 were searched in eight academic databases: PubMed, Ovid Medline(R), EMBASE, PsychInfo (ProQuest), Scopus, SportDiscus, Scielo, and Lilacs. Each search was structured to include two collections of terms: the first age-related (child* in English, criança* in Portuguese, and niño* in Spanish) and the second focusing on aquatic activities (swimming* and aquatic* in English, nadar* and aquático* in Portuguese, and nadar* and acuático* in Spanish).

*Covidence* systematic review management software was used to screen the extracted titles, abstracts, and full texts and to perform the quality assessment. One author (CS) analyzed 100% of the titles and abstracts extracted, and a second author (CBurnay) analyzed 10%; two authors (CS and CBurnay) analyzed the full text of potentially admissible articles. A snowball manual search using the reference list or the citations included in the full-text screening was performed to identify additional articles.

The following data was extracted from the included articles: Study ID (authors and year of publication); participants age and sample size (in experimental and control groups); country; study design; outcome domain (health, development, and physiological); outcome measures (type, assessment tool, and time point of assessment); and results (effect estimates, effect direction, confidence intervals, etc.).

### Quality Assessment

The quality of all the selected articles was analyzed by one author (CS), and two others (CBurnay and RC) analyzed 50% of the articles each. ROBINS-I tool [23] was used to assess the risk of bias of all the studies, except those with a cross-sectional design; in these cases, the JBI Critical Appraisal Checklist for Analytical Cross-Sectional Studies tool [24] was applied. In cases of disagreement between the authors, a third author (CBurnay or RC) resolved the divergence. Following the procedures of the ROBINS-I tool, a preliminary consideration of confounders was conducted for all the included articles.

The risk of bias due to confounding was analyzed, taking into account information reported about specific conditions that are considered influential on 0–36 months old babies’ development and health (e.g., family incoming status [25], parental education, environmental factors, antenatal care, birth weight, gestational age, birth order, gender of the child [26,27,28], prematurity [26,27,28,29], and mode of delivery [30,31]).

## 3. Results

**Figure 1:**
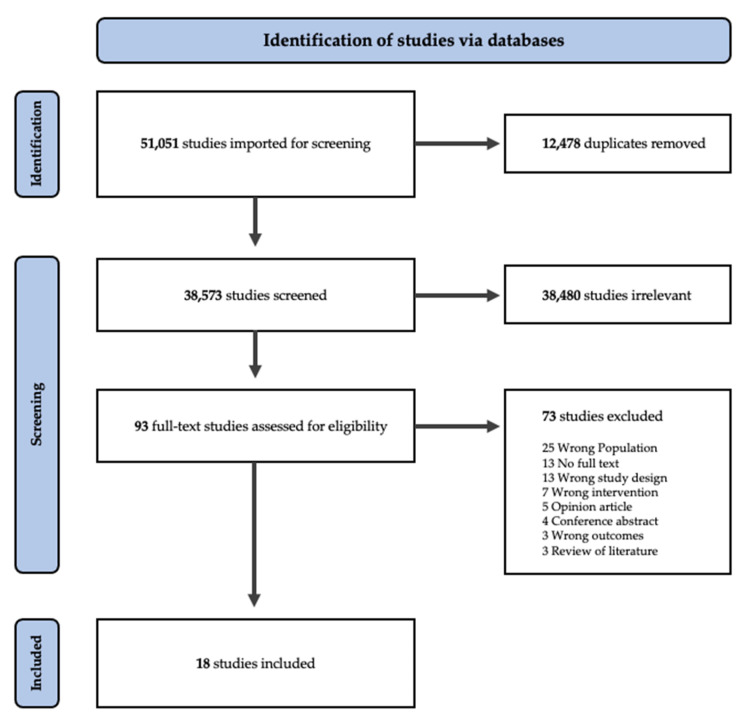
PRISMA flow chart outlining the procedure of extracting articles for inclusion.

Eighteen articles were identified for data extraction in this review (Figure 1). Two of the included studies were assessed through a snowball manual search. In all included studies, the exposure to formal aquatic activities occurred in swimming pools or other types of indoor facilities, and no studies on the exposure of infants to open water/natural environments (e.g., ocean, rivers, and lakes) were found. Of the 18 studies, 12 addressed the exposure of infants to swimming programs (i.e., baby swimming programs), and 6 focused on aquatic therapy programs for infants. To aid the analysis, intervention was clustered in the domains of outcomes–health and development for studies in baby swimming programs and the health, physiological, and developmental domains for the studies focusing on baby aquatic therapy programs. Considering the inclusion criteria, no study was found with drowning prevention as an outcome. Descriptive details of the 18 studies included in this review are in Table 1.

As reported in Table 1, of the 18 identified studies, 15 were analytical, investigating potential relationships between dependent and independent variable(s), and only three studies were descriptive. Thirteen studies were observational, with no manipulation of direct interventions; the remaining five were quasi-experimental, with manipulation of the intervention but no random assignment, and no experimental studies were found. Half of the studies (nine) used a longitudinal design, with multiple (at least two) data collection points. Finally, 13 studies were prospective and 5 were retrospective. In the intervention clusters, studies with samples bigger than 100 were observational and were mainly in the baby swimming health domain. Only one study in the baby swimming developmental domain had a sample size bigger than 30 participants in the intervention group [40]. Half of the studies (nine) had samples smaller than 30 for the intervention group. One-third (six) of the studies included were from Brazil and focused mainly on aquatic therapy interventions, and five studies were from Scandinavian countries, focusing on baby swimming programs.

The main reasons for excluding studies in the full-text screening were the lack of age specificity and the absence of a control group.

### 3.1. Effects of Baby Swimming Programs on Infants’ Health

Table 2 presents the results of the studies focusing on the effect of baby swimming programs attendance on infants’ health.

Two studies reported the risk of diarrhea and/or giardia proliferation and contamination in infant swimming groups [32,34]. The chance of having diarrhea was lower for infants who never attended swimming groups or attended only after the first year of life compared to those who attended the pool earlier [34]. Even in properly treated swimming pools, the participation of children infected with giardia seems to be an agent of contamination to other children attending in swimming programs [32]. In the study reported by Harter et al. [32], none of the children in the control group were contaminated, despite contact outside the pool with children exposed to giardia.

Five studies investigated the effect of swimming pool attendance on infants’ lower respiratory tract infections (LRTI) [33,34,35,36,37], and in all five studies, no evidence of an association was observed. However, infants’ recurrent respiratory tract infections [33] and increased risk of wheezing [35] were associated with atopic parents (i.e., possessing an extremely sensitive form of allergy). Wheezing was associated with human rhinovirus infection (HRV), and it has been shown to be more common among swimming than non-swimming infants [37]. In the cases of respiratory syncytial virus (RSV)-associated wheezing, no significant differences between swimming and non-swimming infants were found [37]. Two studies reported no association between otitis media infections and infants’ participation in swimming programs [33,34]. One study [34] reported more infants with asthma among those who never joined swimming classes or only did it after the first year of age and no association between baby swimming programs attendance and the presence of hay fever and eczema. The same results of no association between eczema and swimming pool attendance were found in a second study [36].

### 3.2. Effects of Baby Swimming Programs on Infants’ Development

Table 3 presents the results for the effect of baby swimming attendance on infants’ development. One pilot study [39] pointed to an increase in developmental percentile (measured using AIMS) after baby swimming program intervention. Similar results were found in cross-sectional studies showing significant improvements in gross [40,41,43], fine, and total motor development [41] in infants participating in swimming programs when compared with the control groups and that four-year-old children who previously participated in baby swimming programs had better scores in prehension (ball skills) and in static balance (one-leg balance) than children that did not participate in baby swimming programs [38]. In cognitive performance, no significant pre- or post-test differences were found in either the intervention or control group, only a marginal tendency towards intervention-related gains in inhibition speed and response selection (or shifting) accuracy [41].

One study [42] used EEG to access visual evoked potentials (VEPs) and measure the functional integrity of the visual pathways from the retina to the visual cortex of the brain addressing infants’ motion perception, which is crucial for a successful navigation through the environments. A greater improvement of motion perception was observed in extra-stimulated infants (i.e., infants that participated in swimming programs) when compared with full-term, traditionally raised peers and preterm infants [42].

### 3.3. Effects of Aquatic Therapy on Infants’ Health and Physiological Parameters

As reported in Table 4, a significant increase in body weight was observed in newborns exposed to aquatic therapy when compared to non-intervention infants [44]. Benefits in the sleep–wake cycle and reduction of pain signals in preterm newborns that were exposed to aquatic therapy were also observed [45,46].

Regarding the physiological parameters, Silva et al. [47] reported lower heart rates on post and follow-up tests compared to baseline measures before submerging infants in a bucket filled with water. Although the intervention was applied twice on alternating days, we only considered between pre-, post-, and follow-up results; between-session results were not considered in this review once no control group was available to confirm the results. Two other studies also reported that newborn heart rates (HRs) reduced 10 to 30 min after aquatic therapy [45,46]. No changes in arterial pressure were observed after aquatic therapy interventions [45].

While two studies pointed toward a significant increase in blood oxygenation after aquatic therapy sessions [45,46], a third study found no significant effect from intervention [47]. Conflicting results were also observed for the effect of aquatic therapy on infants’ body temperature and respiratory rate (RR): one study reported no significant changes in the RR [47], while another study found a decrease in the RR after aquatic therapy intervention [45]; one study reported no changes between pre- and post-exposure in body temperature [45], while another found a decrease in body temperature after aquatic therapy sessions [46].

Two studies investigated the effect of aquatic therapy on the parameters associated with the newborn criteria for discharge (i.e., defecation and characteristics of the meconium) [44,47]. Time to first defecation was significantly lower in the intervention group and meconium turned yellow faster, factors associated with a higher neonatal jaundice avoidance [44].

### 3.4. Effects of Aquatic Therapy on Infants’ Development

Only two studies addressing the effects of aquatic therapy on infants’ development meeting the inclusion criteria were found (Table 5). One study showed a positive effect of aquatic therapy on infants’ functional mobility [48]. The second study reported an increase in the number of infants considered typically developed versus the number of those considered at risk or with delayed development after the aquatic therapy intervention, with the tendency maintained four months after the intervention, revealing an associated learning effect [49]. In this particular study [49], the Affordances in the Home Environment for Motor Development—Infant Scale (AHEMD-IS) score was considered a control variable, but no significant differences were found between groups. An additional analysis of aquatic therapy effects on their quality of life showed some significant improvements in the physical capacity of infants in the intervention group [49]. However, these results should be interpreted with caution, because the assessment tool was validated for the minimum ages of 2 to 4 years old (see [50]), and the sample used was younger than that.

### 3.5. Quality Assessment

The risk of bias assessment is reported in Table 2, Table 3, Table 4 and Table 5. The major problems were related with the risk of bias due to confounding (63%), followed by outcome measurement (26%), exposure measurement (21%), criteria for inclusion in the sample (11%), and statistical analysis (5%). Only three studies (16.6%) presented no risk of bias.

## 4. Discussion

In this systematic review, studies targeting the effects of exposing 0–36-month-old infants to organized aquatic activities on their motor, social, cognitive and emotional development and health and safety were screened and assessed against quality criteria. Initially, over 51,000 sources were identified in the review process and 18 published studies were accepted based upon the inclusion criteria. Two types of studies hatched from the process: studies regarding baby swimming activities and those regarding aquatic therapy. The included studies were clustered in three different domains (health, physiology, and development) to aid the interpretation.

No studies regarding baby SPAs or infant exposures to aquatic environments in natural and ethnographic-related contexts meeting the inclusion criteria were found. Although the design of the review was deliberately broad to identify all possible outcomes arising from exposing infants to aquatic environments, no studies on infants’ safety (i.e., drowning prevention) and social and emotional development meeting the inclusion criteria were found. On drowning prevention, the specific age range of 0 to 36 months had not been addressed in the previous literature, and potentially important studies were not considered in the present systematic review due to their design. For instance, the effects of experience in swimming programs on infants under two years of age on the development of complex swimming competencies were investigated by Zelazo and Weiss [51], but the absence of a control group meant this study could not be included in this review.

### 4.1. Health Domain

Baby swimming has previously been identified as a risk for gastrointestinal tract infections, dermatitis, acute respiratory illness, hyponatremia, and hypothermia, as well as LRTI [14]. However, in the present systematic review, swimming pool attendance was ‘only’ associated with an increased risk of diarrhea [32,34] and a higher chance of wheezing associated with human rhinovirus infection [37]. None of the included studies found any association between baby swimming participation and the diagnosis of otitis media. No clear association was found regarding the health effects of the swimming pool byproducts exposition on LRTI [15,16], except in the particular cases of children with atopic parents [33,35].

In the aquatic therapy context, research has largely focused on the benefits for infants and, in many cases, newborns and premature babies. Since results in the physiological domain indicate that this practice is safe for kids [44,45,46,47], aquatic therapy can be regarded as a facilitative means to achieve newborn discharge criteria and a preventative measure against neonatal jaundice [44]. According to Mitra and Rennie [52], jaundice is a yellow discoloration of the skin and sclera in infants associated with the accumulation of bilirubin in the tissues. In most term babies, jaundice will remain harmless, but high levels of bilirubin in the brain, can lead to a state of toxicity, called ‘kernicterus’, that could result in the infant’s death. Aquatic therapy was also linked to the well-being of premature infants, stimulating pain reduction and better sleep–wakefulness cycles [45,46]. In therapy, the adoption of pharmacologic and nonpharmacologic interventions to reduce pain is crucial due to neonate hypersensitivity to painful stimuli [53]. Preterm newborns can receive a median of 51 painful procedures per day during hospitalization [53]. Full-body interventions such as tucking, swaddling, kangaroo care, and massage therapy have been used for alleviating immediate pain during invasive procedures, but research is lacking on the routine use of these therapies for reducing long-term pain effects [54].

### 4.2. Physiological Domain

Studies on the effect of aquatic therapy interventions on infants’ physiological parameters are important to understand the safety of these practices [47]. However, studies have reported conflicting results, showing that either the intervention does not affect SaO_2_ and RR [47] or it promotes healthier and safer values (i.e., SaO_2_ increase and RR decrease [45,46]). The HR reduction, after the intervention, observed and reported in the included studies [46,47] seems to be linked to a calming effect of aquatic therapy on infants. Regarding the effect of aquatic therapies on infants’ body temperature, the only article meeting the inclusion criteria [46] reported a reduction in temperature, which required ongoing monitoring to avoid a decrease to dangerous values. The observed decrease was, however, considered to be within normal temperature patterns. Aquatic therapy intervention for infants has thus far been reported as a safe practice.

In baby swimming interventions, two potentially relevant studies [55,56] were not included due to the absence either of a control group or baseline measurements in a pre-post-intervention design.

### 4.3. Development Domain

The positive effect on child neurodevelopment reported by Garcia and colleagues in their systematic review [17] was confirmed for infants younger than 36 months in the present review. Although three studies had already been included in Garcia et al.’s review [38,39,48], more recent studies confirmed the positive effects of aquatic activities exposure on infants’ development [41,42,43,49]. Baby swimming programs were associated with improvements in gross, fine, and total motor development [39,40,41,43] (even when the children are older [38]); improvement in motion perception [42]; and a tendency for an improvement in early executive function skills [41].

The present review established a positive association between time of baby swimming attendance and general motor skills improvement. Borioni et al. [41] reported that 10 sessions of baby swimming were enough to increase the motor and cognitive skills of infants. Response shifting, also called cognitive flexibility, is the child’s capacity to shift from one mental set to another, adjusting to changing demands [41]. Despite the small sample size, Borioni and colleagues’ study [41] showed a promising tendency toward intervention-related gains among specific components of executive functioning, suggesting evidence of the benefits of baby swimming on the complex ability to shift between mental sets (cognitive flexibility). Blystad and van der Meer’s results [42] showed significant gains in motor perception in infants that received extra stimulation through baby swimming. The authors pointed out a close link between self-generated actions and the improvements in the optic flow process, indicating that brain maturation is not the only factor leading to the development of visual motion perception. Perhaps, as shown in the classical study by McGraw [6], extra-stimulated infants have more opportunities to interact with their surroundings, possibly becoming more experienced in processing different patterns of visual motion than their peers. Although Sigmundsson and Hopkins [38] did not find a clear association between baby swimming participation and a higher motor development, they did report differences between the experimental and control groups in one-leg balance and ball skills tasks. However, this study needs to be carefully considered, as it was based on parents’ questionnaires, which introduced a potential recall bias. No studies relating 0–36-month-olds aquatic skills acquisition and time of practice were found. Some studies reporting a positive relationship between the number of swimming sessions infants attended and water skills development [57] or time of practice and swimming behavior [51] were analyzed for inclusion. However, these studies were not included in this review due to the lack of control groups.

In the context of aquatic therapy, eight sessions with a twice-a-week periodization significantly increased the number of children with typical development in the intervention group, which did not happen in the control group [49]. All the interventions presented in the analyzed studies involved 45 to 60 min sessions, except in the study of McManus et al. [48], with a 30 min aquatic therapy intervention once a week for 36 weeks. Despite the limitations of McManus et al.’s study [48] (i.e., small sample size, choice of participants with no randomization, lack of generalizability, and a contested measure of functional mobility), the population included children with a wide spectrum of disabilities, such as cerebral palsy, muscular myopathy, chromosomal anomaly, myelomeningocele, sensory system and integration deficits, hypotonia, and developmental delay, providing evidence that the aquatic intervention may be useful across a variety of neuromuscular and developmental delays.

It is important to note that studies considered for analysis in the development domain had a control group that did not receive the aquatic activities intervention, but they also did not receive any other kind of motor intervention. Therefore, the aquatic intervention was an additional time for motor stimulation in the infants’ lives. One specific study by Diem [58] investigated the effect of extra motor stimulation in children, comparing the effects of exposure to baby swimming programs with other types of extra motor stimulation. The results indicated that children who participated in swimming classes during their first year of life benefited from better adaptation to new situations [58]. However, this study did not meet the inclusion criteria, as the report of the assessment tools and the statistical procedures did not guarantee reliability. In future studies, it is important to establish if the motor developmental improvements are the result of motor stimulation in aquatic environments or the result of the any type of extra motor stimulation provided to the child, regardless of the environment. It would also be of interest to further investigate the level of satisfaction experienced by children undergoing aquatic therapy.

### 4.4. Strengths and Limitations of the Review

In the present review, a thorough search for research was conducted by searching eight databases. The recommended systematic review procedures were strictly followed to ensure the rigor of this review; both full-text articles and their study quality were assessed independently by two reviewers. However, a few limitations must be noted. Although no language restrictions were applied when searching for the articles, only keywords in English, Portuguese, and Spanish were used, and only studies in these languages were analyzed. This methodological option might have led to the exclusion of articles in other languages. Additionally, given the large number of articles retrieved, titles and abstracts were only screened by one of the authors. However, we tried to mitigate this limitation by having a second author check 10% of the titles and abstracts.

## 5. Conclusions

The articles included and analyzed in the present systematic review reveal positive effects of exposing infants to baby swimming programs and aquatic therapies on the infants’ motor development, visual motion, and cognitive flexibility. However, due to methodological limitations addressing the impact of baby swimming programs on motor development, we cannot confirm if the results are due to exposing infants to formal aquatic environments specifically rather than to any kind of extra stimulation. Studies comparing groups exposed to different types of motor stimulation, including swimming programs, are needed to ascertain the real effect of infants’ attendance of baby swimming programs on motor development.

Regarding the effect of exposing infants to aquatic environments, although some risk of diarrhea due to the attendance of contaminated swimming pools has been reported, participation in formal aquatic programs has been shown to be a generally safe practice for infants. No association was found between exposure of infants to formal aquatic interventions and lower respiratory track infections or otitis diagnostic. In addition, aquatic therapy programs were beneficial in alleviating a variety of neuromuscular and developmental delays and disabilities, in the prevention of newborns’ jaundice, and other discharge criteria, as well as relieving pain and improvement of the sleep–wakefulness cycle in newborns. The results in the physiological domain in the aquatic therapy context also confirm the safety of the procedures regarding its impact on infants’ heart rate, arterial pressure, respiratory rate, oxygen saturation, and body temperature.

Potentially important studies addressing the effects of exposing infants to aquatic activities were not included due to design biases. Due to the lack of control groups as one of the main reasons for the exclusion of potentially important studies analyzed, the inclusion of a control group is an important recommendation for future studies in this area. Perhaps designs that utilize staggered baselines can help address ethical concerns about infants being allocated to a control group in relation to water safety and drowning.

Some important questions have not yet been properly investigated. For instance, no articles were found regarding baby SPA effects on infants or addressing the effects of any type of infant aquatic exposure on drowning prevention. Studies with infants under 36 months old are needed in these fields. Further consideration of possible confounders, the adoption of procedures to guarantee the reliability of the studies, and better reporting of exposure and measurement procedures are also needed in future investigations.

## Figures and Tables

**Table 1:** Exposure to aquatic environments as an intervention on infants (included studies).

	Domain	Country	1st Author, Year	Age	Intervention Group N	Control Group N	Study Design
**Baby Swimming**	Health	USA	Harter, 1984 [32]	<3 years	70	18	Analytical, Observational, Cross-sectional, Prospective
		Norway	Nystad, 2003 [33]	0–11 months	155	2707	Analytical, Observational, Cross-sectional, Retrospective
		Germany	Schoefer, 2008 [34]	<1 year	660	655/877	Analytical, Observational, Longitudinal, Retrospective
		Norway	Nystad, 2008 [35]	6–18 months	7717	23,152	Analytical, Observational, Cross-sectional, Retrospective
		Spain	Font-Ribera, 2013 [36]	<1 year	1106	1099	Analytical, Observational, Cross-sectional, Retrospective
		Finland	Schuez-Havupalo, 2014 [37]	0–17 months	469	569	Analytical, Observational, Longitudinal, Prospective
	Development	Norway	Sigmundsson, 2010 [38]	2–7 months	19	19	Analytical, Observational, Cross-sectional, Retrospective
		Brazil	Dias, 2013 [39]	7–9 months	6	6	Analytical, Quasi-experimental, Longitudinal, Prospective
		Brazil	Pereira, 2011 [40]	1–18 months	40	40	Analytical, Observational, Cross-sectional, Prospective
		Italy	Borioni, 2022 [41]	0–3 years	12	15	Analytical, Quasi-experimental, Longitudinal, Prospective
		Norway	Blystad, 2022 [42]	<12 months	10	10 + 10 preterm	Analytical, Quasi-experimental, Longitudinal, Prospective
		Italy	Leo, 2022 [43]	6–10 months	14	14	Analytical, Observational, Cross-sectional, Prospective
**Baby Therapy**	Health	China	Zhao, 2005 [44]	Newborns	377	154	Analytical, Observational, Longitudinal, Prospective
		Brazil	Vignochi, 2010 [45]	Newborns	12		Descriptive, Observational, Cross-sectional, Prospective
		Brazil	Novakoski, 2018 [46]	Newborns	22		Descriptive, Observational, Cross-sectional, Prospective
	Physiology	Brazil	Silva, 2017 [47]	Newborns	30		Descriptive, Observational, Longitudinal, Prospective
	Development	USA	McManus, 2007 [48]	6–30 months	15	22	Analytical, Quasi-experimental, Longitudinal, Prospective
		Brazil	Araujo, 2023 [49]	4–18 months	24	37	Analytical, Quasi-experimental, Longitudinal, Prospective

**Table 2:** Effects of exposure to baby swimming programs on infants’ health.

1st Author, Year	Research Aim and Design	Results	Risk of BIAS
**Harter,** **1984 [32]**	**Effect of swimming pool attendance on infants’ (0–3 years of age) giardia diagnosis.** The presence of giardia cysts (in stool sample) was compared between 70 participants in swimming programs (93% of children, 87% of mothers and 36% of fathers) and 18 non-swimming siblings and playmates.	Giardia positivity (G+) for 61% children, 39% mothers and 28% fathers of the swim group; G+ children were found in each of the nine programs swim classes Children G+ for control group = 0% No association between G+ and age (*p* = 0.35) G+ with higher prevalence (71%) for attendance > 7 sessionsG+ prevalence for < 7 sessions = 35%. **Swimming pool attendance increased the odds of infants to become Giardia positive.**	** Confounding Statistical analysis
**Nystad,** **2003 [33]**	**Effect of baby swimming programs attendance on respiratory tract infections and otitis media in the first year of life.** The standard questionnaire of the International Study of Asthma and Allergies in Childhood (ISAAC) was applied to 2862 children 6–16-years-old to access the presence of recurrent respiratory tract infections (RRTI), bronchitis, bronchiolitis, pneumonia, otitis media during the period 0–11 months age. A second questionnaire of parental history of atopy (asthma, eczema or hay fever), demographic information, early exposures and childhood health was applied one year later.	No association between respiratory tract infections and baby swimming. Risk of RRTI [adjusted odds ratio (aOR) 2.08, 95% confidence interval (95% CI) 1.08–4.03] and otitis media (aOR 1.77, 95% CI 0.96–3.25) increases only in children of parents with atopy. **Baby swimming programs increased the RRTI and otitis media in infancy among children of parents with a history of atopy.**	** Confounding Measurement of exposure Outcomes measurement
**Schoefer, 2008 [34]**	**Effect of swimming pool attendance on early infections and development of airway diseases after 1st year of life.** On a 6-year longitudinal study with questionnaires administered to parents on a regular basis (aged 6, 12, 18 months and 2, 4, 6 years), information on socioeconomic factors, medical history (hey fever, asthma, eczema, airway infections, otitis media, diarrhea), and lifestyle factors of 2192 children was obtained. Parental atopy, age of first pool attendance [(a) 1st year baby swimming (N = 660), (b) 1st year occasionally (N = 655) and (c) later or never (N = 877)] and frequency of pool attendance was also accessed.	Non-swimming babies had lower rates of infection of(i) diarrhea: OR = 0.68 (0.54–0.85), CI 95%; (ii) otitis media: OR = 0.81 (0.62–1.05), CI 95%; (iii) airway infections: OR 0.85 CI 95% 0.67–1.09 in the 1st year of life. No clear association between late or non-swimmers and hay fever or atopic dermatitis were found. Higher rates of asthma were found (OR 2.15 95% CI 1.16–3.99), however, potentially due to reverse causation. **Swimming pool attendance increased gastrointestinal infections (i.e., diarrhea) during the first year of life, but no association of swimming pool attendance and atopic diseases and airway infections was found.**	* Confounding
**Nystad,** **2008 [35]**	**Effect of baby swimming in the first 6 months of life on respiratory diseases from 6 to 18 months.** Maternal retrospective report (at 18 months age) about their infants’ lower respiratory tract infections (LRTI), wheeze and otitis media between 6 and 18 months of age (N = 30,870) in the Norwegian Mother and Child Cohort Study (MoBa). History of maternal atopy was also accessed.	LRTI and otitis media were not associated with baby swimming attendance. An increased risk of wheeze [adjusted odds ratios (aOR) 1.24 (95% CI 1.11, 1.39)], on children who attended baby swimming was only observed on children with atopic mothers. **Baby swimming programs increased the likelihood of wheeze in infants with a maternal history of atopy.**	** Measurement of exposure Measurement of condition Outcomes measurement
**Font-Ribera, 2013 [36]**	**Effect of baby swimming programs attendance on respiratory symptoms and infections during the first year of life.** Parent report (at 14 months age) about LRTI, persistent cough, wheezing, otitis and atopic eczema during the first year of life (N = 2205 infants). Swimming pool attendance during the first year of life and parental atopy was also accessed.	Adjusted OR of wheezing [1.06 (95%CI, 0.88–1.28)] and LRTI [1.09 (0.90–1.31)] for babies not attending vs. babies attending swimming pools. Type of swimming pool (indoor or outdoor), and parental atopy did not modify the results. **Swimming pool attendance during the first year of life was not associated with LRTI, otitis, wheezing, atopic eczema or persistent cough.**	** Measurement of exposure Outcomes measurement
**Schuez-Havupalo, 2014 [37]**	**Effect of baby swimming programs attendance on infants’ (0–17 months of age) respiratory tract infections.** Wheezing, bronchiolitis, number of days per year with rhinorrhea, cough or fever recorded. 1827 children were followed up from birth until 17 months of age, on baby swimming attendance, wheezing, bronchiolitis, number of days per year with rhinorrhea, fever or cough. Viral diagnostics were performed in a subset of children with all respiratory tract infections.	An increased likelihood of wheezing illness was observed in swimming children when compared to non-swimming children (*p* = 0.11). Rhinoviruses were more correlated with wheezing in swimming children [11/296 (3.7%)] than non-swimming children [4/339 (1.2%)] (*p* = 0.04). Baby swimming attendance had an odds ratio of 1.71 (*p* = 0.05) for bronchiolitis and 3.57 (*p* = 0.06) for rhinovirus- associated wheezing. Baby swimming attendance was associated with rhinovirus-associated wheezing for children with atopic eczema (*p* = 0.006). **Infant swimming programs increased respiratory tract infections in atopic infants.**	* None

Quality analysis tool: * ROBINS-I and ** JBI.

**Table 3:** Effects of exposure to baby swimming programs on infants’ development.

1st Author, Year	Research Aim and Design	Results	Risk of BIAS
**Sigmundsson,** **2010 [38]**	**Effects of baby swimming programs attendance on infants’ (2–7 months) subsequent motor abilities.** Motor abilities of 19 four-years-old children who attended baby swimming programs during first year of life (mostly between 2 and 7 months of age) were tested using Standardized Movement Assessment Battery for Children and compared with an age-matched control group of 19 who did not attend baby swimming programs.	Performance in prehension, ball skill sub-test (*p* < 0.05), and static balance, one-leg balance sub-test (*p* < 0.017) were better in the swimming group. **Baby swimming programs promote better motor skill development specifically in provision of vestibular stimulation and eye–hand coordination.**	** Confounding Criteria for inclusion in the sample Measurement of exposure
**Dias,** **2013 [39]**	**Effect of baby swimming programs attendance on infants’ (7–9 months of age) gross motor development.** Gross motor skills were accessed using Alberta Infant Motor Scale (AIMS) before and after four months of weekly playful baby swimming classes with babies (N = 6) and compared with a control (N = 6).	Results revealed a difference between pre- and post-tests (*p* < 0.02) for both groups, with Cohen’s *r =* 0.90 in experimental group indicating a larger effect than observed in the control group (Cohen’s *r* = 0.69); and a larger effect size in the experimental group (*r* = 0.47) of the change in comparison to the control group (*r* = 0.06). No differences between groups were observed. **Baby swimming programs attendance facilitated the development of infants’ gross motor skills; however, the sample size was too small to generate significant differences.**	* Confounding
**Pereira,** **2011 [40]**	**Effect of participating in baby swimming programs and program participation period in infants’ (1–18 months of age) motor development.** Motor development, accessed using Alberta Infant Motor Scale (AIMS), was tested on a group of infants who participated in a program of aquatic activities (N = 40), and compared with a matched control group of non-swimmers (N = 40).	Motor development of non-swimming child was lower (Chi^2^ = 16.59; *p* < 0.001). A significant correlation was found (rho = 0.42; *p* = 0.012) between the time attending baby swimming program and percentile values: longer exposure was related with higher percentile values. **Baby swimming program attendance and participation period had positive influence in infants’ motor development.**	** Confounding
**Borioni,** **2022 [41]**	**Effect of baby swimming programs attendance on infant’ (0–3 years of age) motor and cognitive development.** Peabody Developmental Motor Scale was applied to assess 0–3 years old gross motor skills (GM), fine motor skills (FM) and total motor skills (TM), and core tests of executive functions was applied to access Cognitive development (delayed response for working memory, object retrieval for inhibition and A-not-B for response shifting) before and after 10 weekly 45 min sessions of baby swimming intervention (N = 12), as well as control group (N = 15).	Motor development: For the intervention group, post-test of GM, FM, and TM scores were higher than pre-test scores (GM: *Z* = −2.98, *p* = 0.003; FM: *Z* = −2.97, *p* = 0.003; TM: *Z* = −3.08, *p* = 0.002). For the control group no significant different between pre and post-test were observed. GM, FM, and TM scores were higher for intervention group (GM: *U* = 35.50, n1 = 12, n2 = 15, *p* = 0.006; FM: *U* = 25.50, n1 = 12, n2 = 15, *p* = 0.001; TM: *U* = 25.00, n1 = 12; n2 = 15, *p* = 0.001). Cognitive performance: No differences between pre and post-test were found in either group. A marginal significant change in inhibition speed (*Z* = −2.12, *p* = 0.034), response shifting accuracy (*Z* = −1.87, *p* = 0.062) and in perseveration errors (*Z* = −2.00, *p* = 0.046) were observed on intervention group (given the adjusted *p* < 0.016 for three comparisons). **Baby swimming programs attendance may benefit motor development and early executive function skills.**	* Confounding
**Blystad,** **2022 [42]**	**Effect of extra motor stimulation in the form of baby swimming on development of visual motion perception during first year of life.** Brain responses to visual motion, accessed using EEG recordings and onset of self-produced locomotion (documented with parental video records) were obtained on a longitudinal study design at the ages of 4–5 months and 9–12 months on infants that received extra stimulation in the form of baby swimming (N = 10), infants that received a traditional Western upbringing (N = 10), and preterm infants (N = 10). Infants were presented with visual motion on a large screen simulating forward optic flow, reversed optic flow, and random visual motion.	Infants receiving extra motor stimulation and infants in the control group showed developmental improvements in visual motion perception, with a greater improvement for intervention group. Extra-stimulated infants also showed significantly shorter N2 latencies for visual motion and started to locomote at a younger age than the control and preterm groups. **Baby swimming programs attendance during first year of life promotes accelerated developmental improvements of visual motion perception.**	* Confounding
**Leo,** **2022 [43]**	**Effect of baby swimming programs on infants’ (6–10 months of age) motor development** Motor development was assessed using Peabody Developmental Motor Scale-2 in a group of infants attending baby swimming programs (N = 14) and a control group (N = 14).	Better scores on measures of reflexes (*t* = −2.2, *p* < 0.05), grasping (*t* = −3.8, *p* < 0.001), fine-motor quotient (*t* = −3.4, *p* < 0.01), and total-motor quotient (*t* = −2.4, *p* < 0.05) were observed in the intervention group. **Baby swimming programs positively influence early motor development in infants and toddlers.**	** Measurement of exposure

Quality analysis tool: * ROBINS-I and ** JBI.

**Table 4:** Effects of exposure to aquatic therapy on infants’ health and physiological parameters.

1st Author, Year	Research Aim and Design	Results	Risk of BIAS
**Zhao, 2005 [44]**	**Effect of neonatal swimming necklace (water therapy) during hospitalization on newborns’ clinical parameters.** Clinical parameters (weight before discharge, time of first defecation, meconium turning yellow) were recorded via daily monitoring in newborns exposed to aquatic exercises helped by nurse, twice/day for 10–15 min using neonatal swimming necklace (N = 223) and control group who received normal bathing (N = 154).	Weight at discharge: Spontaneous vaginal delivered infants (IG = 3.29 ± 0.35 kg; CG = 3.09 ± 0.38; *p* < 0.01); Caesareans delivered infants (IG = 3.51 ± 0.40 kg; CG = 3.17 ± 0.48; *p* < 0.01). Time of first defecation: Spontaneous vaginal delivered infants (IG = 7.03 + 4.80 h; CG = 8.53 + 5.06; *p* < 0.05); Caesareans delivered infants (IG = 6.54 + 3.59 h; CG = 8.13 + 4.16; *p* < 0.05) Time of meconium turning yellow: Spontaneous vaginal delivered infants (IG = 39.15 + 15.88 h; CG = 48.01 + 19.42 h; *p* < 0.01); Caesareans delivered infants (IG = 39.02 + 13.60 h; CG = 55.67 + 25.05; *p* < 0.05). **Neonatal swimming necklace therapy promoted babies’ growth, earlier onset of first defecation and onset of meconium turning yellow in the early stage.**	* None
**Vignochi, 2010 [45]**	**Effects of aquatic therapy on pain, sleep cycle and wakefulness on preterm infants.** Sleep-wakefulness cycle, assessed using the adapted Brazelton scale, pain, assessed by the occurrence of signs of pain according to the Neonatal Facial Coding System (NFCS) scale, blood pressure; body temperature; heart rate (HR); oxygen saturation (SaO_2_); respiratory rate (RR) (assessed using a Dixtal brand monitor) were measured on 12 preterm infants before, during, at the end, after 30 min, and after 60 min of being placed in a liquid medium for aquatic physical therapy lasting 10 min. Movements to stimulate flexor posture and postural organization were performed.	Sleep-wakefulness cycle: before intervention = 6; during intervention = 4; end of intervention = 3; 30 min after = 1.5; 60 min after = 1 (*p* < 0.001). Pain: Compared with baseline, the mean of pain measure decreased during the intervention (*p* = 0.012), at the end, after 30 and 60 min (*p* < 0.001). No significant differences for mean blood pressure and body temperature before to after intervention. HR and RR were significantly lower (*p* = 0.001 and *p* < 0.001) and SaO_2_ significantly higher (*p* < 0.001) comparing baseline with 30 and 60 min after intervention. **Aquatic therapy reduced pain and improved sleep quality in preterm infants.**	* Confounding Outcomes measurement
**Novakoski, 2018 [46]**	**Effects of aquatic physiotherapy on physiological variables, sleep disturbances, wakefulness, and pain on preterm infants.** Pain, assessed using the Neonatal Facial Coding System (NFCS) scale; sleep state and wakefulness, accessed using adapted Brazelton scale; heart rate (HR) and oxygen saturation (SaO_2_) (recorded using a pulse oximetry); body temperature (recorded using G-Tech digital thermometer), were obtained in 22 preterm newborns at three moments: 5 min before intervention, immediately after (assessment 2) and 10 min after intervention (assessment 3). Infants were wrapped in soft fabric and immersed at shoulder level in warm water in a standard plastic bucket. Sideways, forward, backward and rotational movements were performed.	Pain reduction was observed between evaluation moments: before intervention = 3.68 ± 0.25; assessment 2 = 1.04 ± 0.12; assessment 3 = 0.40 ± 0.12 (*p* = < 0.001). Sleep and wakefulness improvement between evaluation moments: before intervention = 4.45 ± 0.30; assessment 2 = 3.54 ± 0.19; assessment 3 = 2.81 ± 0.21 (*p* = < 0.05). Body temperature decreased from first evaluation (36.52 °C ± 0.62 °C) to assessment 2 (36.24 ± 0.07 °C, *p* < 0.01); but was maintained from assessment 2 to assessment 3 (36.22 ± 0.06 °C, *p* = 1.0). HR rates decrease between first evaluation (154.27 ± 2.6 bpm) and third evaluation (143.72 ± 3.38 bpm, *p* = 0.003). SaO2 increased between evaluation 1 (94.50% ± 0.60%) and evaluation 2 (97.31% ± 0.36%, *p* = 0.001); gains were maintained in evaluation 3 (97.86% ± 0.33%). **Aquatic therapy was effective in improving sleep, wakefulness and physiological parameters and reducing pain in preterm newborns**.	* Confounding Outcomes measurement
**Silva, 2017 [47]**	**Effects of bucket aquatic therapy on physiological parameters in preterm newborns.** Thirty preterm newborns were submerged in a bucket with warm water, up to the height of clavicles, during 10 min in two sessions in alternated days. Heart rate (HR), measured using a wrist oximeter; respiratory rate (RR), assessed through observation of the movements of the rib cage for one-minute counting on an analogue clock; and oxygen saturation (SaO_2_), measured using a wrist oximeter, were accessed three times per session: pre-intervention (15 min before the aquatic therapy), post-intervention (immediately after aquatic therapy) and follow-up test (30 min after post-intervention).	A significant reduction of HR between pre-test (152.23 ± 3.13) and follow-up test (146.53 ± 2.92) was observed (*p* < 0.05). No significant differences for RR and SaO_2_ between assessment moments was observed. **Bucket aquatic therapy with warm water decreased HR in hospitalized premature newborns.**	** Confounding

Quality analysis tool: * ROBINS-I and ** JBI.

**Table 5:** Effects of exposure to baby aquatic therapy programs on infants’ development.

1st Author, Year	Research Aim and Design	Results	Risk of BIAS
**McManus, 2007 [48]**	**Effect of aquatic therapy on functional mobility in 6–30 months infants with delayed functional mobility.** Functional mobility, measured using Gross Motor Subscale of the Mullen Scales of Early Learning (MSEL), was accessed in 15 infants diagnosed with delayed functional mobility before and after receiving 36 weekly 30 min aquatic therapy sessions in a pediatric pool in addition to 60 min home-based early intervention with a physical therapist or occupational therapist. A randomly selected comparison group (N = 22) received home-based early intervention only.	Age-adjusted normalized scores increased in the intervention group (baseline: 27.5 ± 9.8; follow-up: 30.5 ± 11.2; mean change score: 2.6 ± 9.3 points) and decreased in the control group (baseline: 32.5 ± 9.6; follow-up: 28.4 ± 10.1; mean change score: −2.6 ± 8.7 points). The intervention group had significantly greater (*p* < 0.05) gains in functional mobility than the comparison group. **Aquatic therapy improved infants’ functional mobility (gross motor development).**	* Confounding Criteria for inclusion in the sample
**Araujo, 2023 [49]**	**Effect of Kids Intervention Therapy–Aquatic Environment (KITE) program on 4–18-months-old infants’ neuropsychomotor development.** Alberta Infant Motor Scale (AIMS) and Denver II Developmental Screening Test were initially applied to assess and stratify sample as well as at the end of the intervention and after four weeks (to assess retention). Regarding initial characteristics, neonatal and gestation characteristics, and family characteristics; children were classified as Typical or At-risk and delayed. Paediatric Quality of Life Inventory™ Infant Scales (PedsQL™) and Affordance in the Home Environment for Motor Development–Infant Scale (AHEMD-IS) were applied to both groups at the same 3 moments: before intervention, after 4 weeks of intervention and 4 weeks after. Intervention group (N = 24) received four weeks of aquatic environment therapy: 45–60 min, twice a week, of fun aquatic activities (Kids Intervention Therapy–Aquatic Environment (KITE) program); Control group (N = 37) had no intervention besides the daycare center participation.	In the intervention group, number of typical children increased at post-intervention (*p* = 0.001) and retention (*p* = 0.002), with a large intervention effect (η2 = 0.178 and 0.156) and delayed/at-risk cases decreased in post-intervention test, with a medium intervention effect in IG (η2 = 0.055). The intervention group had a significant medium effect of QOL on intragroup physical capacity at post-intervention (*p* = 0.023). No significant differences between the groups, at baseline, in QOL and in home stimulation were observed. No significant change in CG throughout the research. **Fun aquatic activities had positive effects on typical and delayed/at-risk infants’ neuropsychomotor development, motor learning through retention and on QOL physical capacity domain.**	* None

Quality analysis tool: * ROBINS-I.

## Data Availability

Not applicable.
